# Comprehensive Evaluation of the 5XFAD Mouse Model for Preclinical Testing Applications: A MODEL-AD Study

**DOI:** 10.3389/fnagi.2021.713726

**Published:** 2021-07-23

**Authors:** Adrian L. Oblak, Peter B. Lin, Kevin P. Kotredes, Ravi S. Pandey, Dylan Garceau, Harriet M. Williams, Asli Uyar, Rita O’Rourke, Sarah O’Rourke, Cynthia Ingraham, Daria Bednarczyk, Melisa Belanger, Zackary A. Cope, Gabriela J. Little, Sean-Paul G. Williams, Carl Ash, Adam Bleckert, Tim Ragan, Benjamin A. Logsdon, Lara M. Mangravite, Stacey J. Sukoff Rizzo, Paul R. Territo, Gregory W. Carter, Gareth R. Howell, Michael Sasner, Bruce T. Lamb

**Affiliations:** ^1^Department of Radiology and Imaging Sciences, Indiana University School of Medicine, Indianapolis, IN, United States; ^2^Stark Neurosciences Research Institute, Indiana University School of Medicine, Indianapolis, IN, United States; ^3^The Jackson Laboratory, Bar Harbor, ME, United States; ^4^Department of Medicine, University of Pittsburgh, Pittsburgh, PA, United States; ^5^The Jackson Laboratory for Genomic Medicine, Farmington, CT, United States; ^6^Sage Bionetworks, Seattle, WA, United States; ^7^Department of Medicine, Indiana University School of Medicine, Indianapolis, IN, United States; ^8^Department of Medical and Molecular Genetics, Indiana University School of Medicine, Indianapolis, IN, United States

**Keywords:** Alzheimer’s disease, phenotyping, animal model, early-onset AD, MODEL-AD

## Abstract

The ability to investigate therapeutic interventions in animal models of neurodegenerative diseases depends on extensive characterization of the model(s) being used. There are numerous models that have been generated to study Alzheimer’s disease (AD) and the underlying pathogenesis of the disease. While transgenic models have been instrumental in understanding AD mechanisms and risk factors, they are limited in the degree of characteristics displayed in comparison with AD in humans, and the full spectrum of AD effects has yet to be recapitulated in a single mouse model. The Model Organism Development and Evaluation for Late-Onset Alzheimer’s Disease (MODEL-AD) consortium was assembled by the National Institute on Aging (NIA) to develop more robust animal models of AD with increased relevance to human disease, standardize the characterization of AD mouse models, improve preclinical testing in animals, and establish clinically relevant AD biomarkers, among other aims toward enhancing the translational value of AD models in clinical drug design and treatment development. Here we have conducted a detailed characterization of the 5XFAD mouse, including transcriptomics, electroencephalogram, *in vivo* imaging, biochemical characterization, and behavioral assessments. The data from this study is publicly available through the AD Knowledge Portal.

## Introduction

Alzheimer’s disease (AD) is the leading cause of dementia, and there are currently no effective intervention strategies to prevent, slow, or reverse the disease process (Citron, 2010; Karch et al., 2014). The number of people affected by AD in the US is expected to rise to 13.8 million by 2050, and the worldwide financial burden of the disease is projected to reach $9.12 trillion in the same time-frame (Jia et al., 2018), highlighting the critical need for the development of novel therapeutics (Hebert et al., 2013). The primary characteristics of AD are extracellular parenchymal β-amyloid (Aβ) plaque formation and intracellular accumulation of hyperphosphorylated tau in neurofibrillary tangles (NFTs), which lead to significant synaptic impairment, inflammation, and progressive cognitive decline (Citron, 2010; Karch et al., 2014). Late-onset AD (LOAD), in which symptoms typically arise after age 65, is the most common form of AD and accounts for approximately 98% of cases (Isik, 2010). Early-onset AD (EOAD, also known as familial AD or FAD), accounts for ∼2% of AD cases, arises at ages <65, and results from a single inherited mutation, typically in the amyloid precursor protein (APP), Presenilin 1 (PSEN1), or Presenilin 2 (PSEN2) genes (Dai et al., 2018).

Transgenic animal models have been critical for understanding the development and progression of AD (Jankowsky and Zheng, 2017). Owing to the similarities in disease presentation between sporadic LOAD and inherited EOAD and the lack of understanding of the genetic causes of LOAD, known EOAD mutations, such as those observed in APP and PSEN1/2 have been used to develop transgenic mouse models for the study of both LOAD and EOAD (LaFerla and Green, 2012). While transgenic models have been instrumental in understanding AD mechanisms and risk factors, they are limited in the degree of characteristics displayed in comparison with AD in humans, and the full spectrum of AD effects has yet to be recapitulated in a single mouse model (Dodart et al., 2002; Duyckaerts et al., 2008; Porquet et al., 2015; Jankowsky and Zheng, 2017). This incomplete development of AD pathology in EOAD models likely contributes significantly to the difficulty in translating therapeutics developed in EOAD mice to humans in clinical trials to date. Despite promising advances, no effective treatment has been established for AD (Cavanaugh et al., 2014; Cummings et al., 2014, 2019).

The Model Organism Development and Evaluation for Late-Onset Alzheimer’s Disease (MODEL-AD) consortium was assembled by the National Institute on Aging (NIA) to develop more robust animal models of AD with increased relevance to human disease, standardize the characterization of AD mouse models, improve preclinical testing in animals (Sukoff Rizzo et al., 2020), and establish clinically relevant AD biomarkers, among other aims toward enhancing the translational value of AD models in clinical drug design and treatment development (Oblak et al., 2020). The MODEL-AD consortium creates and comprehensively characterizes novel mouse models to help researchers select the most appropriate mouse for their therapeutic studies and provide relevant measures and protocols to assay AD-like phenotypes in appropriate therapeutic windows (Oblak et al., 2020). It is hoped that the development of more clinically appropriate AD mice and a more in-depth understanding of the phenotype of existing AD mouse models available to the AD research community will facilitate improved translatability from mouse models to human clinical trials.

To this end, the Indiana University/Jackson Laboratory/University of Pittsburgh (IU/JAX/Pitt) MODEL-AD Center has conducted an exhaustive phenotyping study of the commonly used 5XFAD mouse model to establish and validate a characterization pipeline that will ultimately be used to comprehensively phenotype new mouse models of AD. The 5XFAD transgenic mouse was developed in 2006 and overexpresses human *APP* with three FAD mutations [the Swedish (K670N, M671L), Florida (I716V), and London (V7171) mutations] and human *PSEN1* with two FAD mutations (M146L and L286V) (Oakley et al., 2006). The expression of both transgenes is regulated by neural-specific elements of the mouse *Thy1* promoter to drive their overexpression specifically in brain neurons (Moechars et al., 1996). Understanding the phenotypic changes in the 5XFAD mouse enhances the utility of the model for developing clinical interventions, including enhanced appreciation of its limitations and the time point(s) at which therapeutics can be appropriately evaluated in this model in the context of translatability.

Here, the IU/JAX/Pitt MODEL-AD Center presents a systematic and comprehensive phenotypic analysis of the 5XFAD mouse congenic on the C57BL/6J (B6) background at age 4, 6, and 12 months with a comparison between male and female mice relative to age-matched controls, including the characterization of regional Aβ deposition, proinflammatory marker expression, aging-related frailty, metabolic health (level of cholesterol, lipoprotein, and glucose), behavioral testing response, gene expression via RNA-seq, and brain electrical activity via electroencephalogram (EEG) analysis. All data and standard operating procedures (SOPs) are available through the AD Knowledge Portal^1^.

## Materials and Methods

### 5XFAD Transgenic Mouse Model

5XFAD transgenic mice overexpress the following five FAD mutations: the APP(695) transgene harbors the Swedish (K670N, M671L), Florida (I716V), and London (V7171) mutations, and the PSEN1 transgene harbors the M146L and L286V FAD mutations. The 5XFAD line was made congenic on the C57BL/6J background in 2011 to minimize concerns related to allele segregation and the high variability of the original hybrid background. 5XFAD mice exhibit amyloid deposition, gliosis, and progressive neuronal loss accompanied by cognitive and motor deficiencies, recapitulating many of the features of human AD. NFTs are not typically present in the 5XFAD model (Oakley et al., 2006), however, indicating a significant difference between the AD pathology of this model and human disease.

### Animal Housing Conditions at Indiana University and the Jackson Laboratory

Male hemizygous 5XFAD mice (MMRRC stock #: 34848) were crossed with female C57BL6/J mice (JAX stock #: 000664) to maintain the 5XFAD and non-transgenic WT colonies at Indiana University and The Jackson Laboratory. Up to five mice were housed per cage with Aspen SaniChip bedding and *ad libitum* LabDiet^®^ 5K52/5K67 (6% fat) feed. The colony room was kept on a 12:12 L:D schedule with the lights on from 7:00 am to 7:00 pm daily. Transgenic and WT mice were initially ear-punched for identification; following genotyping, mice were identified with p-chip (PharmaSeq) microchips placed at the base of the tail. Both male and female littermate mice aged 4, 6, and 12 months old were used for this study. These ages reflect early disease state (4 months), moderate disease state (6 months), and late disease state (12 months). In all cases, non-transgenic littermates were used as controls. All procedures were approved by the Institutional Animal Care and Use Committees at the Indiana University, The Jackson Laboratory, and the University of Pittsburgh.

### Animals and Housing Conditions at the University of Pittsburgh

Adult male and female 5XFAD mice (MMRRC stock #: 34848) and non-transgenic C57BL/6J controls (JAX stock #: 000664) were received from The Jackson Laboratory at approximately 8 weeks of age. Mice were group-housed within sex and genotype (*n* = 3–4 per cage) prior to surgical procedures, after which the animals were single-housed to prevent damage to the EEG implants by other cagemates. The housing rooms within the AAALAC-certified vivarium at the University of Pittsburgh consisted of ventilated caging and an automated watering system with room temperature controlled at a setting of 22 ± 2°C and humidity at 50 ± 10%. The facility was on a 12:12 L:D schedule (lights on at 7:00 am). Housing cages contained coarse certified Aspen Sani-chip bedding (PJ Murphy, Mount Jewett, PA, United States) and enrichment in the form of nestlets (Ancare Corp., Bellmore, NY, United States) and translucent domes (Braintree Scientific Inc., Braintree, MA, United States). Lab Diet 5P76, irradiated chow (Lab Diet, St. Louis, MO, United States) was available *ad libitum* throughout all procedures.

### Surgical Procedures for EEG Studies at the University of Pittsburgh

Mice between 10 and 11 months of age, two-channel telemetric transmitters [HD-X02; Data Sciences International (DSI), New Brighton, MN, United States] were subdermally implanted near the dorsal aspect of the rear flank. Each channel consisted of two wire leads that run under the skin to the head and neck of the mouse. Animals were anesthetized using isoflurane in an anesthetic chamber (Compac 5, VetEquip, Livermore, CA, United States), hair was shaved from the top of the head, and ophthalmic ointment (Artificial Tears, generic) was applied to the eyes before the mice were secured in a stereotaxic frame (Model 940, David Kopf Instruments, Tujunga, CA, United States) equipped with a gas anesthesia nosecone (Kopf 923B) and rubberized zygoma ear cups (Kopf 921). Body heat was regulated using delta-phase isothermal pads (Braintree Scientific, Braintree, MA, United States). A stable plane of anesthesia was maintained throughout the procedure and verified at least every 15 min by the toe-pinch reflex. Once the mice were secured, the skin over the dorsal aspect of the shoulders to behind the eyes was sanitized using iodine and isopropyl alcohol before making a dorsal midline incision from just behind the eyes to the scapulae. The exposed skull was completely cleared of blood and tissue and further cleaned with 3% hydrogen peroxide. This cleaning process is critical to ensure the bonding of the dental acrylic used to the secure wire leads to the skull. For cortical EEG, two small burr holes were drilled at AP: +1.0, ML: ±1.0 and AP: −2.0, ML: ±1.0, with ML coordinates opposing, counterbalanced by animal. The ends of the wires were stripped, and one lead was placed through each burr hole so as to touch, but not penetrate, the dura. The wires and skull were then covered in glass ionomer dental acrylic (Integrity A2, Dentsply Sirona, Charlotte, NC, United States). For neck electromyography (EMG), stripped wires from the second channel were inserted under the neck muscle and secured on each side by non-absorbable sutures (Ethilon Black Monoflament, Ethicon Inc., Somerville, NJ, United States). The animal was then removed from the stereotaxic frame and the incision was closed with non-absorbable sutures and sterilized with triple-antibiotic ointment. Fifteen minutes prior to the end of the procedure, animals received a subcutaneous 5 mg/kg dose of carprofen for prophylactic analgesia. The animals were left to recover on an isothermal heating pad in a clean home cage until the righting reflex was restored, at which time the animals were returned to the vivarium. Animals all received daily post-operative carprofen for a minimum of 48 h or as needed to treat acute pain. No animals in this cohort required post-operative carprofen for more than 72 h. Sutures were removed 7–9 days post-operatively.

### EEG Recording Procedures

Electroencephalogram recordings took place 12–14 days following implantation. Cohorts of eight mice, divided by sex, were recorded in separate sessions 1 week apart, with males recorded in cohort 1 and females in cohort 2. Each session was recorded continuously over 72 h, beginning and ending in the afternoon. Animals were brought into the testing room 1 h before recording procedures began. Wire chow holders were removed from the cages to allow for unobscured video monitoring, with chow and HydroGel (Clear H2O, Portland, ME, United States) available *ad libitum* in the cage. Implants were activated in each mouse and the home cage was placed on top of the DSI receiver before the recordings began. EEG and EMG data were sampled continuously at 500 Hz, and activity and temperature were sampled at 1 Hz using DSI Ponemah software. Each recording was time-locked to a video camera interfaced with Noldus Media Recorder software (Noldus, Leesburg, VA, United States). Lights in the testing room were timed such that only red light was present between 7 pm and 7 am to allow for video recording during the animals’ inactive (dark) period. Animals were left undisturbed during the recording session aside from daily health and food monitoring.

### EEG Quantification and Statistical Analysis

Analysis of spike-wave activity was conducted using DSI NeuroScore software. Recordings were initially band-pass filtered between 0.5 and 80 Hz with a power-line filter at 60 Hz. Epochs of seizure-related spike-wave discharge were autonomously identified by the software according to the following parameters: dynamic threshold: 3–20 times baseline, spike duration: 1–200 ms spike interval: 0.05–0.6 s, train duration minimum: 0.5 s, train Join Interval: 1 s, minimum four spikes per train associated with behavioral arrest in the video feed. Sleep stages were quantified using SleepSign for Animals (Kissei-Comtec, Matsumoto City, Nagano Prefecture, Japan) by comparing the delta (0.5–4 Hz) and theta (4–8 Hz) power ratio during epochs where EMG activity was below a threshold set for each animal according to its baseline (generally 30–50 μV), indicating the absence of tone in the neck muscles, and activity counts were zero. A delta ratio above 0.4 during inactive EMG was quantified as slow-wave sleep (SWS), whereas a theta ratio above 3 during these epochs was quantified as paradoxical sleep. Power band distribution values were derived using fast Fourier transform during each wake-sleep stage. Values were plotted, and effects of genotype and sex were analyzed by ANOVA with Tukey’s multiple comparison tests using Prism 8 statistical software (GraphPad, San Diego, CA, United States). A significance level of *p* < 0.05 was used for all measures.

### Perfusion and Preparation of Blood and Tissue Samples

Mice at Indiana University and The Jackson Laboratory were anesthetized to the surgical plane of anesthesia with tribromoethanol at 4, 6, or 12 months of age. Under complete anesthesia, animals were euthanized by decapitation and perfused through the heart with ice-cold PBS. Trunk blood and brain tissue were collected immediately after euthanasia. Trunk blood was centrifuged for 15–20 min at 4°C at 14,500 RPM, and the plasma was stored at −80°C. At the University of Pittsburgh, mice were anesthetized with isoflurane and blood was collected followed by decapitation.

The right brain was snap-frozen for the preservation of RNA for gene expression studies. Total RNA was extracted using Trizol (Invitrogen, CA, United States), and mRNA was purified using biotin-tagged poly dT oligonucleotides and streptavidin-coated magnetic beads. Quality was assessed using a NanoDrop (Fisher Scientific).

### Blood Plasma Analysis

Blood was collected from non-fasted mice aged 4, 6, or 12 months. Mice were anesthetized, and blood was extracted by left ventricle cardiac puncture with a 25 g EDTA-coated needle before PBS perfusion. Approximately 500 mL of whole blood was transferred to a MAP-K2 EDTA Microcontainer (BD, Franklin Lakes, NJ, United States) on ice and centrifuged at 4°C at 4388 × *g* in a pre-chilled ultracentrifuge for 15 min. Without disturbing the red blood cell fraction, serum supernatant was pipetted into a chilled cryovial with a p200 tip and immediately snap-frozen on dry ice for 10 min. Samples were stored at −80°C and later thawed for analysis of glucose, total cholesterol, low-density lipoprotein (LDL), high-density lipoprotein (HDL), triglyceride, and non-essential fatty acid (NEFA) levels with the Siemens Advia 120 (Germany).

### Tissue Vision Whole-Brain Imaging and Quantification of Plaque Progression

Mice were given an intraperitoneal injection of methoxy-X04 (5 mg/kg) to label amyloid plaques. Twenty-four hours after injection, mice were euthanized by injection of ketamine/xylazine (0.1 mL/10 g) and sequentially perfused with 1× PBS and 4% PFA. Intact brains were dissected and post-fixed in 4% PFA for 12–24 h at 4°C. Whole-brain serial two-photon (STP) tomography imaging was performed using a TissueCyte 1000 (TissueVision, Inc.) (Ragan et al., 2012). Brain samples were imaged at 780 nm with 220 mW measured at the objective, and emitted fluorescence was captured across three channels (ch1: 560–680, ch2: 500–560, and ch3: 400–500 nm). Methoxy-X04 fluorescence was predominantly identified in channel 3. One hundred and forty serial block-face images were acquired at 100 μm spacing for each brain at ∼1.4 μm/pixel XY sampling. Automated segmentation of the fluorescent signal from methoxy-X04 labeled plaques across all sections was performed by local threshold-based segmentation. The relative signal intensity between the tissue autofluorescence signal identified in channel 1 and the methoxy-X04 signal identified in channel 3 was first computed by channel subtraction to remove the local background autofluorescence and identify the methoxy-X04 signal. A manually defined threshold was then applied to the background-subtracted methoxy-X04 signal to minimize false positives and generate a plaque mask for each section. This background subtraction also reduced broad-spectrum false positive signal intensities identified across all three channels often identified around the olfactory bulbs. The imaging and segmentation parameters were held constant across all samples and all ages (2 months – eight females, eight males; 3 months – seven females, eight males; 4 months – eight females, six males; 6 months – eight females, five males). To map brain regional plaque densities, the Allen Brain Reference Atlas was registered onto each brain sample using the Allen Mouse Brain Common Coordinate Framework v3 (CCFv3) (Wang et al., 2020). Briefly, the 10 μm average template brain from the CCF was registered to a 10× down-sampled version (14 μm × 14 μm × 100 μm XYZ) of each brain sample using a multi-resolution coarse-to-fine linear to non-linear series of transformations (Ragan et al., 2012; Kuan et al., 2015), and the corresponding Atlas Annotations were then registered using the computed transform matrix. The masked plaque density (fractional area) was mapped for each brain region across sections by computing the ratio of total masked pixels to total region pixels across all sections after up-sampling of the atlas annotation section to the full resolution STP datasets.

### Spatial Proteomics Using NanoString GeoMx Spatial Profiling

Hemibrains were embedded in paraffin and sectioned at 8 μm. Staining was carried out according to the manufacturer protocol. Microglia, astrocytes and plaques were labeled with IBA-1, GFAP and pan-amyloid antibodies, respectively (NanoString, Seattle, WA, United States). Nuclei were identified by staining with the nucleic acid-binding fluorescent dye, SYTO 13 (Thermo). Amyloid plaques were selected by visual inspection. Using NanoString’s GeoMx^TM^ Digital Spatial Profiler system to immunostain for AD, PD, autophagy and glial cell relevant proteins, amyloid-positive plaques were identified in the cortex, hippocampus, and cerebellum. These selected areas were illuminated individually via UV light on GeoMx DSP, photocleaving the oligonucleotides present within each region of interest (ROI). The oligonucleotides were collected on GeoMx DSP in a 96-well microwell plate. Individual microwells containing the collected photocleaved oligonucleotides from each spatially resolved ROI were then hybridized to four-color, six-spot optical barcodes, and analyzed on the nCounter^®^ platform, resulting in distinct spatially mapped counts that correspond to the antibodies from which the tags were cleaved. Digital counts were normalized with internal controls for system variation and then normalized to area and background.

### Homogenization and Protein Extraction

Each hemibrain was weighed prior to homogenizing in tissue protein extraction reagent (T-PER; Thermo Scientific; 1 mL per 100 mg of tissue weight) supplemented with protease and phosphatase inhibitors cocktail (Sigma-Aldrich). Total protein concentration was measured using bicinchoninic acid (BCA; Pierce). Hemibrain lysates were then aliquoted and kept in the −80°C freezer for long-term storage. Following tissue homogenization, the DEA extraction of the soluble components was performed as previously reported (Casali and Landreth, 2016). The primary supernatant was utilized to analyze the content of proinflammatory cytokines, while the soluble DEA components were used for the Aβ species analysis.

### Cytokine Panel Assay

Mouse hemibrain samples were assayed in duplicate using the MSD Proinflammatory Panel I (K15048D; MesoScale Discovery, Gaithersburg, MD, United States), a highly sensitive multiplex enzyme-linked immunosorbent assay (ELISA). This panel quantifies the following 10 proinflammatory cytokines in a single small sample volume (25 μL) of supernatant using an electrochemiluminescent detection method (MSD): interferon γ (IFN-γ), interleukin (IL)-1β, IL-2, IL-4, IL-6, IL-8, IL-10, IL-12p70, IL-13, and tumor necrosis factor α (TNFα). The mean intra-assay coefficient for each cytokine was <8.5%, based on the cytokine standards. Any value below the lower limit of detection (LLOD) for the cytokine assay was replaced with 1/2 LLOD of the assay for statistical analysis.

### Aβ Species Assay

The plasma samples and soluble (DEA) components from hemibrain samples were assayed in duplicate using MSD Aβ Peptide Panel I (K15200E; MSD). Following the manufacturer’s guidelines, the Aβ species (Aβ40 and Aβ42) were quantified from plasma and soluble component of hemibrain lysates using an electrochemiluminescent detection method (MSD). The levels of Aβ species from the assay were used for statistical analysis.

### Magnetic Resonance Imaging

To obtain high-contrast gray matter images, at least 2 days prior to PET imaging, mice were induced with 5% isoflurane in medical oxygen, placed on the head coil, and anesthesia was maintained with 1–3% isoflurane for scan duration. High-resolution T2-weighted (T2W) magnetic resonance imaging (MRI) images were acquired using a 3T Siemens Prisma clinical MRI scanner outfitted with a dedicated 4-channel mouse head coil and bed system (RAPID MR, Columbus, OH, United States). Images were acquired using a SPACE3D sequence using the following acquisition parameters yielding 0.18 mm × 0.18 mm × 0.2 mm resolution images: TA: 5.5 min; TR: 2080 ms; TE: 162 ms; ETL: 57; FS: On; Ave: 2; Excitation Flip Angle: 150; Norm Filter: On; Restore Magnetization: On; Slice Thickness 0.2 mm: Matrix: 171 × 192; and FOV: 35 mm × 35 mm. After the imaging period, mice were returned to their warmed home cages and allowed to recover.

### Radiopharmaceuticals

Regional brain glycolytic metabolism was monitored using 2-[18F]-fluoro-2-deoxy-D-glucose ([18F] FDG), which was synthesized, purified, and prepared according to established methods (Yu et al., 2006). Similarly, (E)-4-(2-(6-(2-(2-(2-([18F]-Fluoroethoxy)ethoxy)ethoxy)pyridin-3-yl)vinyl)-*N*-methyl benzenamine ([18F]-AV45) was synthesized, purified, and prepared according to established methods (Yao et al., 2010). In all cases, clinical unit doses ranging from 185 to 370 MBq (5–10 mCi) were purchased from PETNet Indiana (PETNET Solutions Inc.).

### *In vivo* Imaging

Mice were anesthetized with 5% isoflurane gas and maintained at anesthetic plane with 1–3% isoflurane throughout imaging. Mice were injected IV with 3.7–11.1 MBq (100–300 uCi) [18F]-AV45, and IV or IP with [18F]-FDG at a volume <10% of body weight and allowed 30 min of uptake in an isothermal cage. In all cases, mice receiving [18F]-FDG were fasted for a minimum of 12 h prior to tracer administration. Mice were scanned on the IndyPET3 (Rouzes et al., 2004) scanner for 15 min for both [18F]-AV45 and [18F]-FDG. Calibrated, decay, and scatter-corrected PET images were reconstructed into a single-static image volume with a minimum field of view of 60 mm using filtered-back-projection according to published methods (Soon et al., 2007), and converted from native NetCDF to an Analyze 12-compatible format via a local bash script prior to analysis.

### Cryotomy

After PET imaging, animals were euthanized via rapid decapitation, and brains were removed, bisected, and snap-frozen on dry ice before embedding in OCT in cryomolds. Brains were sectioned at 20 μm with a Leica 1850 or CM1860 Cryotome, with 6 per slide per bregma ROI. The bregma targets of 0.38, −1.94, −3.8, −5.88 based on stereotactic mouse brain coordinates (Franklin and Paxinos, 2013) were selected to cover the following ROIs: Corpus Callosum, Striatum, Cingulate Cortex, Motor Cortex, Somatosensory Cortex, Dysgranular Insular Cortex, Medial Septum, Hypothalamus, Hippocampus, Retrosplenial Dysgranular Cortex, Auditory Cortex, Dorsintermed Entorhinal Cortex, Thalamus, Temporal Association Cortex, Visual Cortex, and Cerebellum. One half of the brain is utilized for autoradiography, the other half is used for IHC as a tertiary confirmation.

### Autoradiography

Standards for calibration were prepared using twofold serial dilutions of PET tracer in 3.2% low melt agarose, 4% sucrose, 0.2% gelatin. Aliquots (∼30 μL) were dotted into cryomolds and allowed to solidify at room temperature. OCT compound was added, and standards were frozen and cut at the same thickness as the brain sections. An additional ∼20 μL aliquot was pipetted into a 7 mL scintillation tube filled with scintillation fluid and measured on a Beckman LSC 6500 for 15–30 s using the full spectrum window. These data were used to calculate the standard curve for each animal and were converted to Bq/mL according to laboratory SOPs. Brain sections mounted on slides were placed on cardboard along with the standards and exposed overnight to the phosphorimager screen before imaging on the GE Typhoon FLA7000IP.

### Image Analysis

All PET and MRI images were co-registered using a ridged-body mutual information-based normalized entropy algorithm with 9° of freedom and mapped to stereotactic mouse brain coordinates using Analyze 12 (AnalyzeDirect, Stilwell, KS, United States). Post-registration, 56 bilateral regions were extracted via brain atlas and averaged to yield 56 unique volumes of interest (27 bilateral regions) that map to the following key cognitive and motor centers: Agranular Insular Cortex; Auditory Cortex; Caudate Putamen; Cerebellum; Cingulate Cortex; Corpus Callosum; Dorsolateral Orbital Cortex; Dorsintermed Entorhinal Cortex; Dysgranular Insular Cortex; Ectorhinal Cortex; Fornix; Frontal Association Cortex; Hippocampus; Lateral Orbital Cortex; Medial Orbital Cortex; Parietal Cortex; Parietal Association Cortex; Perirhinal Cortex; Prelimbic Cortex; Primary Motor Cortex; Primary Somatosensory Cortex; Retrosplenial Dysgranular Cortex; Secondary Motor Cortex; Secondary Somatosensory Cortex; Temporal Association Cortex, Thalamus; Ventral Orbital Cortex; and Visual Cortex. For autoradiographic analysis, tracer uptake was quantified on hemi-coronal sections by manually drawing regions of interest for 17 regions of interest (namely, Auditory Cortex, Caudate Putamen, Cerebellum, Cingulate Cortex, Corpus Callosum, Dorso-intermediate Entorhinal Cortex, Dysgranular Insular Cortex, Ectorhinal Cortex, Hippocampus, Hypothalamus, Medial Septum, Primary Motor Cortex, Primary Somatosensory Cortex, Retrosplenial Dysgranular Cortex, Temporal Association Cortex, Thalamus, and Visual Cortex) on a calibrated phosphor screen at bregma 0.38, −1.94, and −3.8 mm using MCID (InterFocus Ltd.). Standardized Uptake Value Ratios (SUVR) relative to the cerebellum were computed to permit dose and brain uptake normalization for PET and autoradiograms for each subject, genotype, and age as follows:


(1) SUVR(s,R,g,a)=(R(s,g,a))/(C(s,g,a))

where, s, g, a, R, and C are the subject, genotype, age, region/volume of interest, cerebellum region/volume of interest. Principal component analysis was performed to provide data reduction for all PET and autoradiography regions. Consensus regions of interest were selected, which explained 80% of the variance observed across all regions studied. In all cases, PCA selected regions/volumes of interest were analyzed for differences with time and genotype using a two-way ANOVA (Prism, GraphPad Inc.) where *p* < 0.05 was considered statistically significant.

### Behavioral Tests

Behavioral tests were conducted in the Mouse Neurobehavioral Phenotyping Facility at The Jackson Laboratory as previously described (Sukoff Rizzo et al., 2018) with a minimum of a 1–2 day rest period between tests that were conducted in the following order: frailty assessment with core body temperature recording, open field test, spontaneous alternation, rotarod, and home cage wheel running. On each test day, subjects were transported from the adjacent housing room into the procedure room, tails were labeled with a non-toxic permanent marker with the assigned subject ID number for ease of identification, and subjects were left to acclimate to the testing environment for a minimum of 60 min before testing. Between subjects, all testing arenas were sanitized with 70% ethanol solution and dried prior to introducing the next subject. Lighting in the testing rooms was consistent with the housing room (∼500 lux), except for the cognitive tests, which were conducted under ambient lighting (∼30–50 lux). Wheel running was conducted under standard housing conditions with a 12:12 L:D cycle. At a minimum of 5 days after the conclusion of behavioral testing, tissue was harvested as per MODEL-AD protocols (see text footnote 1).

#### Home Cage Wheel Running

Subjects were individually housed with a wireless running wheel (Med Associates Inc., Fairfax, VT, United States; Cat# ENV-047) and left undisturbed for three consecutive nights except for daily welfare checks. Data were analyzed in 1 min epochs and calculated as the cumulative time spent running and the average total distance traveled over 30 min periods.

#### Frailty Assessment

The frailty assessment was conducted as previously described (Sukoff Rizzo et al., 2018) to assess the spectrum of aging-related characteristics and determine if 5XFAD mice demonstrated alterations in normal healthy aging relative to controls. Following acclimation to the testing room as described above, subjects were individually evaluated for the absence, presence, and severity of 26 characteristic traits and reflexes and scored as 0, 0.5, or 1 by a trained observer who was blind to genotype/age. The following were assessed: alopecia, loss of fur color, dermatitis/skin lesions, loss of whiskers, coat condition, piloerection, cataracts, eye discharge/swelling, microphthalmia, nasal discharge, rectal prolapse, vaginal/uterine/penile, diarrhea, vestibular disturbance, vision loss by visual placing upon the subject being lowered to a grid, menace reflex, tail stiffening, impaired gait during free walking, tremor, tumors, distended abdomen, kyphosis, body condition, breathing rate/depth, malocclusions, and righting reflex. Subjects were evaluated for each measure independently and a cumulative score of all measures was calculated with a maximum score of 26 (frailty index). Normal healthy aging is indicated by age-dependent increases in the cumulative frailty score.

#### Core Body Temperature

Core body temperature was recorded before the conclusion of the frailty assessment with a glycerol-lubricated thermistor rectal probe (Braintree Scientific product# RET 3; measuring 3/4″ L0.028 dia.0.065 tip) inserted ∼ 2 cm into the rectum of a manually restrained mouse for approximately 10 s. The temperature was recorded to the nearest 0.1°C (Braintree Scientific product# TH5 Thermalert digital thermometer). Reductions in core body temperature are typical with aging and inversely correlate with frailty score, indicating morbidity. Increases in body temperature relative to controls may indicate hyperthermic responses induced by stress or inflammation.

#### Open Field Test

Versamax Open Field Arenas (40 cm × 40 cm × 40 cm; Omnitech Electronics, OH, United States) were housed in sound-attenuated chambers with lighting consistent with the housing room. Following acclimation to the testing room as described above, mice were placed individually into the center of the arena, and infrared beams recorded distance traveled (cm), vertical activity, and perimeter/center time. Data were collected in 5 min intervals for 60 min.

#### Spontaneous Alternation

Mice were acclimated to the testing room under ambient lighting conditions (∼50 lux). A clear polycarbonate y-maze (in-house fabricated; arm dimensions 33.65 cm length, 6 cm width, 15 cm height) was placed on top of an infrared reflecting background (Noldus, Netherlands), surrounded by a black floor-to-ceiling curtain to minimize extra-maze visual cues. Mice were placed midway in the start arm (A) facing the center of the y for an 8-min test period. The sequence of entries into each arm was recorded via a ceiling-mounted infrared camera integrated with behavioral tracking software (Noldus Ethovision XT). Percent spontaneous alternation was calculated as the number of triads (entries into each of the three different arms of the maze in a sequence of three without returning to a previously visited arm) relative to the number of alteration opportunities.

#### Rotarod Test for Motor Coordination

An accelerating rotarod (Ugo-Basile; model 47600) was used for this test. Lighting in the testing room was consistent with the housing room. Following acclimation to the testing room as described above, mice were placed on the rotating rod (4 RPM), which accelerated up to 40 RPM over 300 s. Each mouse was subjected to three consecutive trials with an approximate 1-min inter-trial interval to clean the rod between trials. Latency to fall (seconds) was measured. Subjects that fell upon initial placement on the rod before acceleration started were scored as 0 s for that trial.

### Analysis of Behavioral Data

Data were quality-controlled blind prior to analysis to exclude data from mice that could not be tested or failed to meet inclusion criteria as noted above, or data unavailable due to equipment failures and escape episodes. No subjects were considered mathematical outliers or excluded based on mathematical determination (e.g., 2 SD ± mean). Data were analyzed under coded genotypes (A, B, C, etc.) within sex, as one-way or two-way ANOVA as appropriate versus sex- and age-matched WT controls.

### RNA-Seq

The RNA from a total of 36 5XFAD and 36 littermate control mice was sequenced; 6 mice per sex, genotype, and timepoint. Sequencing libraries were constructed using TruSeq DNA V2 (Illumina, San Diego, CA, United States) sample prep kits and quantified using qPCR (Kapa Biosystems). The mRNA was fragmented, and double-stranded cDNA was generated by random priming. The ends of the fragmented DNA were converted into phosphorylated blunt ends. An “A” base was added to the 3′ ends. Illumina^®^-specific adaptors were ligated to the DNA fragments. Using magnetic bead technology, the ligated fragments were size-selected, then a final PCR was performed to enrich the adapter-modified DNA fragments since only the DNA fragments with adaptors at both ends will amplify.

#### Sequencing

Libraries were pooled and sequenced by the Genome Technologies core facility at The Jackson Laboratory. All 36 samples were sequenced on an Illumina HiSeq 4000 using HiSeq 3000/4000 SBS Kit reagents (Illumina), targeting 30 million read pairs per sample. Samples were split across multiple lanes for Illumina HiSeq; once the data were obtained, the samples were concatenated to produce a single file for paired-end analysis.

#### Data Processing

RNA-Seq data were processed with our parallelized and automatic pipeline. Reads were quality-trimmed and filtered using the Trimmomatic tool. Reads passing the quality filtering were mapped to the mm10 reference genome augmented with transgene sequences using the RNA-seq aligner STAR (Dobin et al., 2013). To quantify human *APP* and *PSEN1* transgene expression, we created a chimeric mouse genome by concatenating human *APP* (Chromosome 21: 25880550–26171128) and *PSEN1* (Chromosome 14: 73136418–73223691) gene sequences into the mm10 genome (Ensembl Genome Reference Consortium, build 38) as separate chromosomes (labeled chromosomes 21 and 22). Subsequently, we added gene annotations for *hAPP* and hPSEN1 genes in the resulting gtf file. A STAR index was built from this mouse chimeric genome sequence for alignment, and STAR-generated coordinate-sorted BAM files were mapped to the chimeric mouse genome using this index. The HTSeq-count package was used to obtain raw read counts, and the RSEM software package was used to estimate expression levels for all genes as transcripts per million (TPM) (Dobin et al., 2013). Genes with zero counts in more than 75% of samples were filtered out before applying the upper quantile normalization method to the TPM, which were log-transformed for downstream analysis.

#### Principal Component Analysis

We analyzed 72 RNA-Seq samples originating from the 5XFAD transgenic and B6 non-carrier littermate control mice of different ages. First, we implemented the variance stabilizing transformation (VST) function of DESeq230 to normalize the raw read count data. We then used the biplot function in R to assess the differences in sample distributions due to genotype, age, and sex.

#### Differential Expression Analysis

Differential expression in mouse models was assessed using the Bioconductor package DESeq2 (v1.16.1) (Love et al., 2014). DESeq2 takes raw read counts obtained from HTSeq-count as the input. The significance of differential expression was determined by the Benjamini–Hochberg corrected *p*-values. The threshold for significance was set to adjusted *p* < 0.05.

#### Generalized Linear Model

A generalized linear model (GLM) was used to identify transcripts with differential expression as a function of sex, genotype, and sex-genotype interaction for each age group. The expression value of a gene E in log2 (TPM) was defined using the following linear model:


(2) E=β_0+β_1Sex+β_2Genotype +β_12Sex.Genotype+ε,

where β_0 and ε represent average expression level for the reference WT male mice and residual, respectively.

We used False Discovery Rate (FDR)-corrected *p*-value (*q*-value) and *R*^2^ from the linear model to determine how well each gene’s expression across the samples could be fit by the predictors of the model, which were sex, genotype and sex-genotype interaction. For each gene, the GLM returned an estimate for each predictor’s effect size and corresponding significance. We used a relatively permissive threshold (*q*-value < 0.25 and *R*^2^ > 0.3) to identify transcripts potentially fit by the model. Taking the WT as the reference genotype and male as the reference sex, we extracted significant transcripts (*p* < 0.05) for genotype (5XFAD), sex (female) and sex-genotype interaction (5XFAD female) for each age group.

#### Functional Enrichment Analysis

Functional annotations and enrichment analysis were performed using the R package clusterProfiler (Yu et al., 2012), with Gene Ontology and KEGG pathway enrichment analyses performed using the enrichGO and enrichKEGG functions, respectively. The function compareCluster was used to compare enriched functional categories of each gene module. The significance threshold for all enrichment analyses was set to 0.05 using Benjamini–Hochberg-adjusted *p*-values.

#### Comparison to Human Transcriptomes

Mouse transcriptomes were systematically compared to human disease signatures from the Accelerating Medicines Partnership in Alzheimer’s Disease (AMP-AD) studies (Wan et al., 2020). We computed Pearson correlation between change in expression (log fold change) for each gene in a given module with each mouse model, sex, and age (Wagner et al., 2019; Preuss et al., 2020). Correlation coefficients were computed using the cor.test function built in R as follows:


(3) Cor.test(Log2FC(h),Log2FC(m))

where Log2FC(h) is the log fold change in transcript expression of human AD patients compared to control patients and Log2FC(m) is the log fold change in expression of mouse transcripts compare to age and sex-matched control mouse models. Log2FC values for human transcripts were obtained through the AD knowledge portal.

## Results

### Cholesterol, Lipoprotein, and Glucose Changes in 5XFAD Mice

**FIGURE 1:**
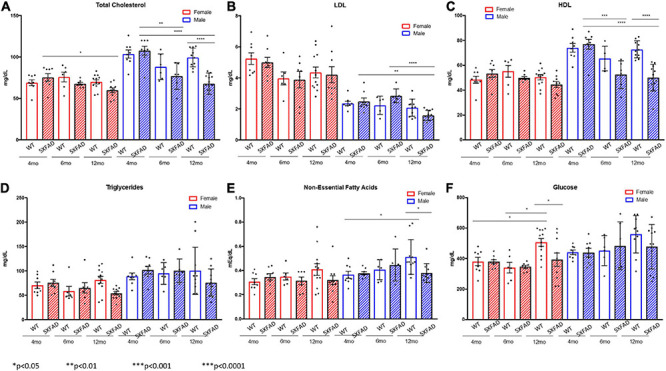
Decreased total cholesterol and lipoproteins in 5XFAD males compared to wild-type males and decreased glucose levels in 5XFAD females compared to wild-type females. Non-fasted plasma was collected at the termination of the study, and total cholesterol **(A)**, low-density lipoproteins **(B)**, high-density lipoproteins **(C)**, triglycerides **(D)**, non-essential fatty acids **(E)**, and glucose **(F)** levels were measured.

Non-fasted plasma was collected at the termination of the study from male and female 5XFAD and WT mice aged 4, 6, and 12 months. Cholesterol levels, LDLs, HDLs, triglycerides, non-essential fatty acids, and glucose levels were compared (Figures 1A–F). Total cholesterol was lower in female 5XFAD and WT mice than in male counterparts at all ages; however, a significant difference in female total cholesterol was present only between 4- and 12-month-old 5XFAD mice (Figure 1A). In male mice, total cholesterol in 5XFAD versus WT mice was not significantly different at 4 months of age, trended lower in 5XFAD mice at 6 months of age and was significantly lower in 5XFAD mice at age 12 months of age (Figure 1A). Female 5XFAD and WT mice had higher LDL levels (Figure 1B), and lower HDL levels (Figure 1C) than males, with no significant difference observed between WT and 5XFAD in females in either lipoprotein. In male mice, LDL and HDL levels were significantly lower at 12 months of age than at 4 and 6 months of age (Figures 1B,C) and HDL levels decreased significantly between WT and 5XFAD mice at the 6- and 12-month time points (Figure 1C). No significant differences were observed in triglyceride levels across all tested groups (Figure 1D). At 12 months of age, WT male mice demonstrated elevated non-essential fatty acids in comparison with 4-month-old WT mice, and non-essential fatty acid levels were markedly decreased in male 5XFAD at the same age (Figure 1E). Finally, glucose levels in female WT mice were significantly elevated between the 4- and 12-month-old groups, which was mitigated in the 12-month-old 5XFAD mice (Figure 1F).

### Hyperactivity Was the Most Robust Behavioral Phenotype in 5XFAD Mice

**FIGURE 2:**
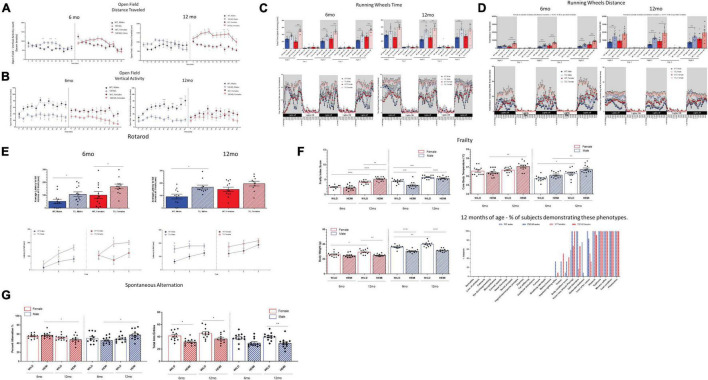
Functional assessment reveals hyperactivity associated with changes in frailty score in 5XFAD mice. Hyperactivity was observed in 5XFAD mice compared with controls was determined using open field and running wheels **(A–D)**. There were increases in distance traveled, vertical and horizonal activity and reductions in resting time. In the rotarod test **(E)**, both male and female 5XFAD mice maintained their balance for a greater period of time than the age-and sex-matched WT controls. Male and female 5XFAD mice and controls demonstrated aging-dependent increases in frailty scores from 6 months to 12 months of age **(F)**. 5XFAD male or female mice did not demonstrate deficits in percent alternation relative to age-and sex-matched controls at either 6 or 12 months of age **(G)**.

In the open-field test, both male and female 5XFAD mice demonstrated increases in total distance traveled and corresponding reductions in total resting time, indicative of hyperactivity (Figure 2A). This phenotype was observed both at 6 and 12 months of age. Interestingly rearing behavior, as measured by vertical activity counts, was significantly reduced in 5XFAD mice relative to the controls, which may be a consequence of increased total distance traveled, a measure analyzed by horizontal activity (Figure 2B). While the open-field test is limited to spontaneous activity in a novel environment for only a 60 min period, hyperactivity was also more robustly reflected during home-cage wheel-running assessments, which were conducted over the course of a 72-h period (Figure 2C). Notably, both male and female 5XFAD mice demonstrated significant increases in time spent on the wheel and averaged significantly greater distances traveled relative to that of age- and sex-matched WT controls, indicative of hyperactivity (Figure 2D). In the rotarod test (Figure 2E), both male and female 5XFAD mice maintained their balance for a greater period of time than the age- and sex-matched WT controls. This does not indicate that 5XFAD mice have impaired motor coordination, but rather increased latency to fall, which has been previously correlated with hyperactivity phenotypes in mice (Wagner et al., 2019) and is in line with the hyperactivity observed in the open-field and wheel-running assessments in the present study.

### Frailty Changes in 5XFAD Mice

Male and female 5XFAD mice and controls demonstrated aging-dependent increases in frailty scores from 6 to 12 months of age (Figure 2F). Notably, 5XFAD mice at both ages demonstrated reductions in body weight relative to the age- and sex-matched controls, similar to what others have previously found (Jawhar et al., 2012). Reduced body weight is unlikely a result of the weight loss observed in aged and “frail” C57BL/6J mice as other traits associated with sarcopenia were not observed. It is more likely that 5XFAD mice do not gain weight at the same rate as WT controls, which may be related to their robust hyperactivity. While 12 months is not necessarily considered “aged” for mice, WT animals show the expected incremental changes across a spectrum of aging-related traits as early as 12 months of age. Interestingly, while the cumulative frailty score in 5XFAD mice, which reflects the spectrum of subtle aging changes across phenotypes, reflected a modest change at best relative to WT controls; analysis of the percentage of subjects presenting with specific aging traits at 12 months of age that were not observed nor expected in controls revealed observations of tremor in *n* = 4/12 (33.3%) 5XFAD males and *n* = 6/12 (50%) 5XFAD females, and impaired righting reflex in *n* = 4/12 (33.3%) 5XFAD males and *n* = 1/12 (8.33%) 5XFAD females at 12 months of age (Figure 2F). Although these phenotypes were not 100% penetrant in 5XFAD mice, they indicate an exacerbated aging phenotype of disease-progression phenotype in these mice. Notably, core body temperature was elevated in both male and female 5XFAD mice relative to age-matched controls, which may be indicative of a hyperthermic response induced by stress or inflammation, which was observed in this model.

### Lack of Reliable Cognitive Phenotypes in 5XFAD Mice in Spontaneous Alternation Assays

Given the previously reported hyperactivity phenotype in 5XFAD mice (Flanigan et al., 2014) that we have confirmed in the present study, we selectively chose the spontaneous alternation task (Figure 2G) to evaluate hippocampal working memory. This test does not have the high level of motor confounds present in other cognitive tests (e.g., the water maze, novel object, and fear conditioning tests). Furthermore, since cognitive tests in mice have failed to provide translational or predictive utility in the clinic, we limited our resources to spontaneous alternation, which has a minimal false-positive rate for cognitive deficits and has highly consistent baselines across both male and female control C57BL/6J mice (Tucker et al., 2016). Furthermore, alterations in spontaneous alternation have been previously reported (Jawhar et al., 2012). In the present studies, male and female C57BL6/J controls demonstrated the expected range for percent alternation, which was consistent across age and sex (49–52%). 5XFAD male or female mice did not demonstrate deficits in percent alternation relative to age- and sex-matched controls at either 6 or 12 months of age (Figure 2G). While there were significant changes within the 5XFAD phenotype in these cohorts between 6 and 12 months of age, the directionality of those changes was opposite in females and males. Interestingly, both male and female 5XFAD demonstrated reductions in total arm entries at both 6 and 12 months of age relative to age- and sex-matched controls during this 8 min test (Figure 2G), which may be reflective of within-arm perseverative–hyperactive behavior. Importantly, the abnormal activity phenotype did not confound the primary outcome measure of spatial working memory in this test.

### No Emergent Phenotype of Sleep or Seizure Activity in 5XFAD Mice

**FIGURE 3:**
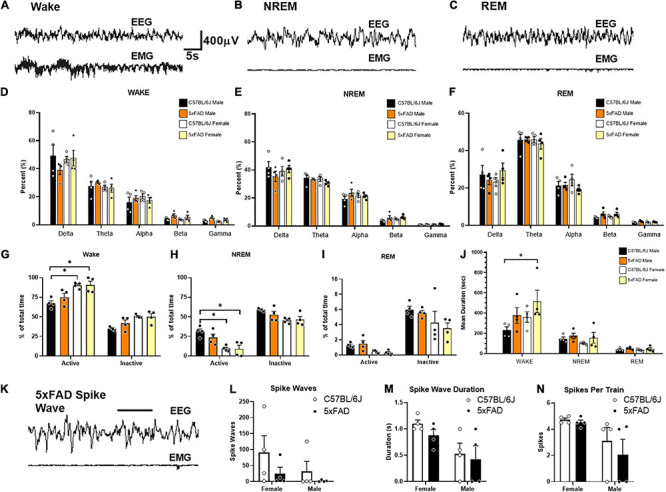
No emergent phenotypes of sleep or spike-wave activity in 5XFAD mice. No emergent phenotypes of sleep or spike wave activity in 5XFAD mice. Sleep and seizure activity was assessed by EEG in female and male 5XFAD mice (*n* = 4 per group). Example traces during epochs of wake **(A)**, NREM sleep **(B)**, and REM sleep **(C)** are displayed across the top. No statistical differences in frequency are measured during wake **(D)**, NREM **(E)**, or REM **(F)**. Analysis of sleep phase distribution during each activity period throughout the 72 h recording does not reveal significant differences in time spent in each respective sleep phase. Increased wake **(G)**, and decreased NREM sleep **(H)** is witnessed in female mice during both activity phases compares to male controls. Differences in REM sleep **(I)** are not seen. Mean duration of wake epochs are significantly increased in females compared to male controls **(J)**. An example of a putative spike wave identified using pike sorting criteria **(K)**. No significant differences in spike wave activity **(L)**, spike wave duration **(M)**, or spikes per train **(N)**, are identified for sex or genotype.

Sleep and seizure activity were compared in male and female 5XFAD mice over a 72-h period (Figure 3), measuring sleep phase distribution (Figures 3A,B) and spike-wave activity (Figures 3C–F) during each activity period. A difference was observed in the distribution of the sleep cycle of female mice during the first inactive period following the movement of the mice to the testing room; however, the sleep phase distribution analysis did not reveal a significant difference between WT and 5XFAD mice of either sex in the time spent in each respective sleep phase overall. No significant difference was observed in spike-wave activity between any of the tested groups.

### Lack of Seizure Activity and Sleep Disturbances in 5XFAD

Overall, no phenotypic differences in EEG seizure markers were identified in 5XFAD mice compared to the littermate controls. No significant differences were found within any frequency band during wake [Figure 3D, *F* (12, 60) = 0.9535], NREM [Figure 3E, *F*(12, 60) = 1.054, *p* = 0.41], or REM sleep [*F*(12, 60) = 0.8458, *p* = 0.60]. C57BL/6J and 5XFAD female mice displayed increased active [*F*(1, 12) = 24.16, *p* < 0.01] and inactive [*F*(1, 12) = 15.99, *p* < 0.01] waking (Figure 3G) and decreased active [*F*(1, 12) = 22.86, *p* < 0.01] and inactive [*F*(1, 12) = 10.29, *p* < 0.01] NREM (Figure 3H). In all mice, these were the main effects and only differed individually compared to C57BL/6J males. A simple main effect of decreased REM sleep was observed in female mice [Figure 3I, *F*(3, 24) = 3.409, *p* = 0.03], but no interaction was observed between individual groups [*F*(3, 24) = 0.4180, *p* = 0.74]. No significant interactions [*F*(6, 36) = 2.039, *p* = 0.09] or main effects [*F*(3, 36) = 2.539, *p* = 0.07] were found for mean episode duration (Figure 3J). *Post hoc* analyses of these trend effects identified an increased wake episode duration in female mice compared to male controls, but no other effects were seen. No significant interaction [*F*(1, 12) = 0.32, *p* = 0.58] or main effect of sex [*F*(1, 12) = 1.64, *p* = 0.22] or genotype [*F*(1, 12) = 2.22, *p* = 0.16] was detected in the number of spike-wave events over the course of the recording. Females exhibited greater spike train duration [*F*(1, 12) = 8.50, *p* = 0.01] and spikes per event [*F*(1, 12) = 6.64, *p* = 0.02] compared to those of males. However, this effect was irrespective of genotype as neither interaction terms for duration [*F*(1, 12) = 0.13, *p* = 0.73] nor spikes per train [*F*(1, 12) = 0.285, *p* = 0.60] differences were significant. No main effects of genotype were detected for spike-wave events [*F*(1, 12) = 2.22, *p* = 0.16], spike train duration *F*(1, 12) = 0.86, *p* = 0.37], or spikes per train [*F*(1, 12) = 0.62, *p* = 0.45].

### Site Is the Greatest Predictor of Microbial Differences

Previous studies have examined the microbiome of AD-related mouse models and found microbial differences. In the present study, we examined the 5XFAD mouse and WT controls over multiple time points (ages 2–21 months) and across three sites. The largest source of variability is site, meaning that the same strains have different microbial profiles depending on where the mice are housed (Supplementary Figures 1A–D). There were few, if any, differences in microbiome composition between 5XFAD and WT within a center when both genotypes were sampled. As this study was a survey of microbiome changes within the 5XFAD mouse, it revealed useful information about center differences and the similarity of 5XFAD and WT animals when comparing animals across sites.

### Genotype and Sex Are a Major Source of Variation in Gene Expression Changes in 5XFAD Mice

**FIGURE 4:**
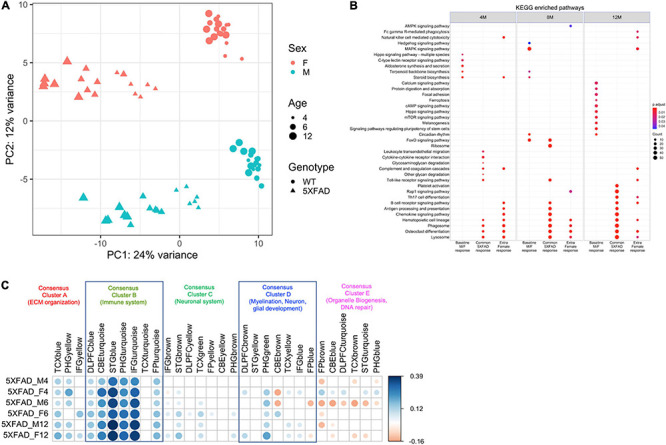
Transcriptomics identified genotype and sex being a major source of variation in between 5XFAD and WT mice and a positive correlation with most of the functionally distinct AMP-AD modules. **(A)** The first principal component accounted for 23% of the total variance and separated 5XFAD samples from WT animals. Female and male samples clustered distinctly at all ages in the second principal component (11% of total variance), suggesting the presence of sex-biased molecular changes in animals. Female and male samples are shown in red and green colors, respectively. 5XFAD and WT controls are represented as circles and triangles, respectively. Increasing point sizes represent the increasing age of the mice (4, 6, and 12 months, respectively). **(B)** KEGG enrichment analysis for genes significantly associated with sex (female), genotype (5XFAD) and sex by genotype interaction (5XFAD female) relative to age-matched male B6 mice. **(C)** Pearson correlation coefficients for gene expression changes in mice (log fold change of the 5XFAD mice minus age-matched B6 mice) and human disease (log fold change for cases minus controls). AMP-AD modules are grouped into five previously identified consensus clusters describing the major functional groups of AD-related alterations. Positive correlations are shown in blue and negative correlations in red. Color intensity and size of the circles are proportional to the correlation coefficient. Correlations with adjusted *p*-value > 0.05 are considered non-significant and are left blank.

Principal component analysis was used to compare the brain transcriptome of male and female 5XFAD and WT mice at 4, 6, and 12 months of age (Figure 4). The first principal component accounted for 23% of the total variance and separated the mice by genotype into the 5XFAD and WT groups. The second principal component accounted for 11% of the total variance and separated male and female mice in both the 5XFAD and WT groups, with each of the four groups clustering distinctly at all ages, indicating sex-based molecular changes in both genotypes (Figure 4A). Furthermore, the KEGG enrichment analysis comparison of male and female 5XFAD and WT mice revealed that genes with significantly upregulated expression in 5XFAD versus WT mice overall had further elevated expression in female 5XFAD mice (Figure 4B).

### 5XFAD Mice Display Transcriptomic Changes Characteristics of AD

Gene expression changes in male and female 5XFAD versus WT mice were compared with changes observed in human patients with AD versus controls using AMP-AD modules grouped into five previously identified consensus clusters that describe the major functional groups of alterations observed in human AD (Wan et al., 2020). Male and female 5XFAD mice display gene expression alterations across all five consensus clusters, with the most pronounced alterations observed in Consensus Cluster B, which consists of immune system pathways (Figure 4C).

### Whole Brain Imaging and Quantification of Plaque Progression

**FIGURE 5:**
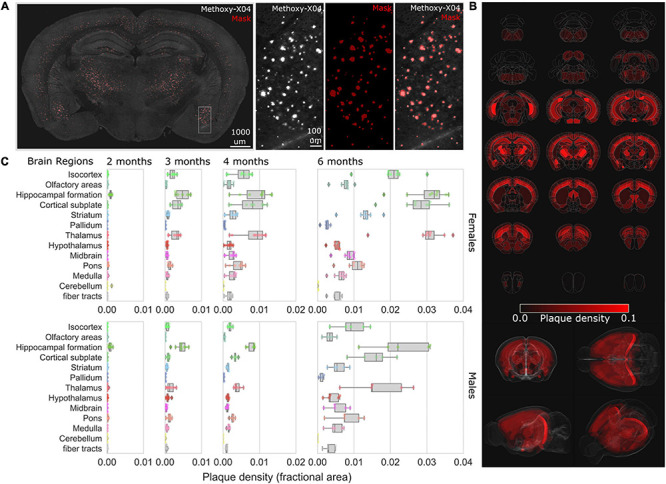
Mapping the progression of methoxy-X04 labeled plaque deposition across brain regions. **(A)** Example brain section from 4-month-old female 5XFAD with methoxy-X04 labeled (gray) and segmentation masked plaques (red overlay). Zoom inset depicts (left to right) methoxy-X04 signal, mask (red), methoxy-X04 signal with mask overlay. **(B)** Plaque densities (fractional area) were quantified for all brain regions depicted as average density heat maps (red) mapped across the entire Allen Mouse Brain Reference Atlas for 6-month-old female 5XFAD, visualized as evenly spaced selected coronal sections (top) and 3D renderings (bottom). **(C)** Box plots of the plaque densities for 13 major brain regions from 2-, 3-, 4-, and 6-month-old female (top) to male (bottom) cohorts demonstrate regional plaque density progression, plots depict median and interquartile range (IQR-25:75) whiskers depict 1.5× IQR, and diamonds for values outside the range.

To map the progression of brain-wide Aβ accumulation in 5XFAD mice, we utilized STP tomography to visualize the spatial distribution of methoxy-X04 labeled Aβ plaques (Klunk et al., 2002) across female and male cohorts at 2, 3, 4, and 6 months of age (Figure 5). Labeled plaques could be readily identified throughout the brain (Figure 5A), and automated analysis routines were developed to segment plaques and map the spatial density across brain regions through registration to the Allen Brain Reference Atlas (Oh et al., 2014; Figure 5B and see section “Materials and Methods”). Labeled plaques were already present at 2 months in both males and females, with the greatest densities present in the hippocampal formation and subcortical structures (Figure 5C), consistent with previous studies (Canter et al., 2019). We observed an age-dependent accumulation in plaque density across brain regions as has been observed in other mouse models of AD (Whitesell et al., 2019). In both females and males, region-specific accumulations were observed, with the greatest densities occurring in the hippocampal formation, cortical subplate, and thalamic regions (Supplementary Table 1).

### 5XFAD Mice Have Increased Glucose Metabolism and Deposition of Aβ as Evidenced by *in vivo* Imaging

**FIGURE 6:**
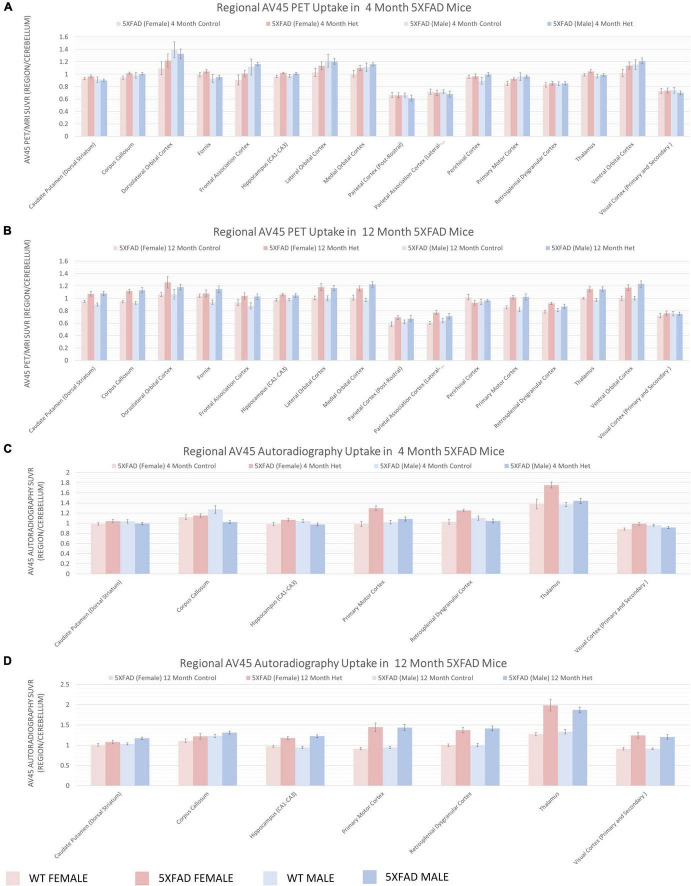
Regional increases in beta-amyloid deposition via [18F]-AV45 PET/MRI and confirmed by Autoradiography. Positron emission tomography (PET) of radiopharmaceutical [18F]-AV45 was used to measure amyloid deposition. PET data presented as brain regions normalized to the cerebellum are quantified in panels **(A,B)** at 4 and 12 months, respectively. Post-mortem autoradiography of coronal brain tissue is represented in panels **(C,D)**.

**FIGURE 7:**
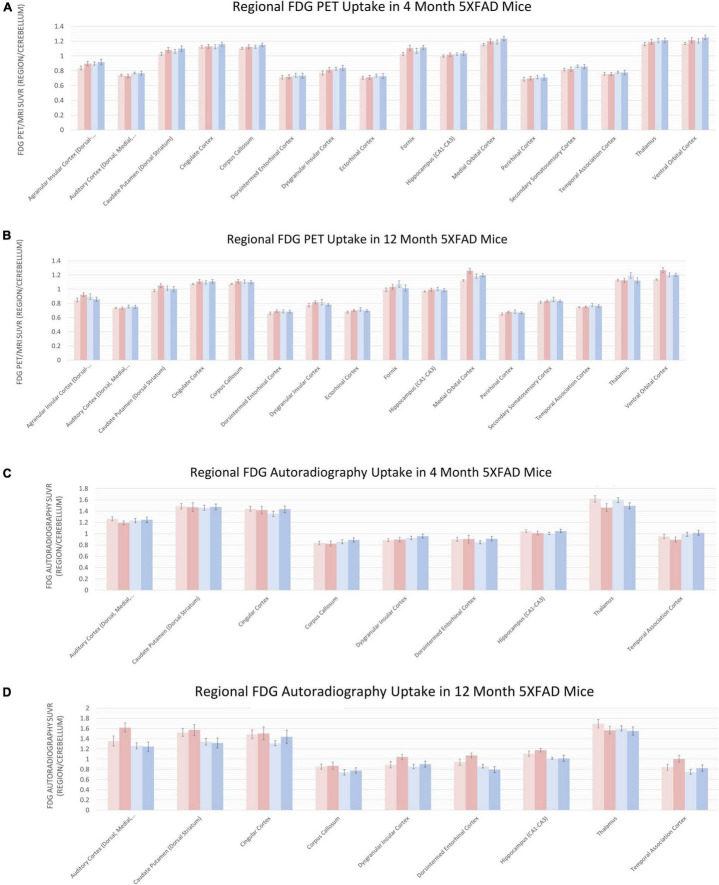
Regional increases in glycolysis via [18F]-FDG PET/MRI and confirmed by Autoradiography. Positron emission tomography (PET) of radiopharmaceutical [18F]-FDG was used to measure tissue glucose uptake. PET data presented as brain regions normalized to the cerebellum are quantified in panels **(A,B)** at 4 and 12 months, respectively. Post-mortem autoradiography of coronal brain tissue is represented in panels **(C,D)**.

To understand regional amyloid deposition (Figure 6) and glycolysis (Figure 7), we performed *in vivo* PET imaging using the [18F] tracers, AV45 and FDG, respectively, similar to what has been done previously in the field (Girard et al., 2013; Tang et al., 2016; Franke et al., 2020; Chang et al., 2021). Amyloid deposition can be observed as early as 4 months in regions involved in emotion and memory (orbital cortex, parietal association cortices), as well as the thalamus and motor cortex using PET (Figure 6A). The amyloid deposition was significantly increased by 12 months of age (Figure 6B). Autoradiography, a measure with 40× greater resolution and 100× greater sensitivity, was used to confirm these findings (Figures 6C,D). Glycolysis, as measured by [18F]-FDG PET, showed little changes at 4 months of age (Figure 7A) and were generally confirmed via autoradiography (Figure 7C); however, in select regions such as the thalamus, [18F]-FDG uptake was decreased 5XFAD mice when compared to WT controls as estimated by autoradiography (Figure 7C). By 12 months of age, reduced glycolysis was observed in both sexes using PET (Figure 7B) and was confirmed via autoradiography (Figure 7D) in male mice, while reduced glycolysis was observed in females in the auditory cortex. As a tertiary measure of the PET imaging data, IHC was completed on the half brains after *in vivo* imaging. ThioflavinS and IBA1 staining (data not shown) confirmed increased microgliosis in the areas with amyloid deposition. Based on these findings, glycolysis was altered in brain regions associated with sensory integration and auditory function in 5XFAD mice when compared with WT controls and was confirmed via post-mortem autoradiography.

### 5XFAD Mice Have Elevated Plasma and Brain Aβ_40_ and Aβ_42_ and Proinflammatory Cytokines

5XFAD mice demonstrate significantly elevated soluble Aβ_40_ and Aβ_42_ levels in the plasma and the soluble components of the hemibrain lysates at 4, 6, and 12 months of age in comparison with the levels observed in WT mice (Supplementary Figure 2A). Additionally, the Aβ42/40 ratio was significantly increased between 4, 6, and 12 months of age in the soluble hemibrain fractions and plasma (Supplementary Figures 2A,B). We also examined the neuroinflammatory response to amyloid deposition and the level of proinflammatory cytokines (IL-1β TNF-α, and KC/GRO) was significantly elevated in 5XFAD hemibrain lysates during disease progression (Supplementary Figure 2C).

### Analysis of the Aβ Plaque Microenvironment Using the Nanostring GeoMx Platform Reveals Differences Between Hippocampus and Cortex

**FIGURE 8:**
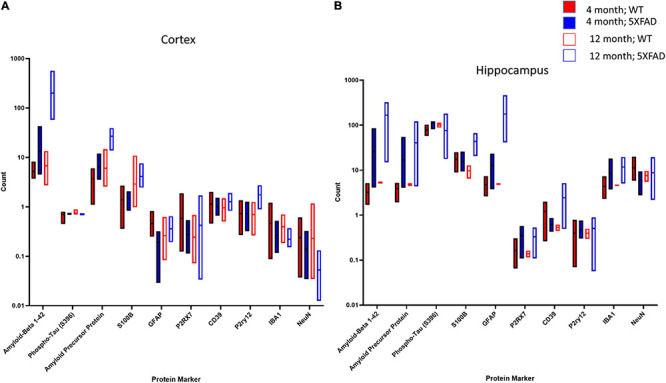
NanoString GeoMX system indicates protein changes in cortex and hippocampus in 5XFAD mice compared to controls at 4 and 12 months. In the cortex **(A)**, several protein markers were upregulated in the 5XFAD mouse compared to controls at both time points. The greatest increases in the cortex were in Aββ1–42 and APP, while reductions were observed in NeuN by 12 months of age. In the hippocampus **(B)**, markers related to AD pathology (Aββ1–42, APP) as well as inflammation were upregulated as early as 4 months of age.

To further identify differences between 4- and 12-month-old 5XFAD mice with respect to the Aβ plaque microenvironment, we took advantage of the NanoString GeoMx platform to obtain a detailed view of >40 proteins in the plaque microenvironment simultaneously with digital quantitation and spatial resolution (Figure 8). We selected the frontal cortex and hippocampus from four mice per experimental group and analyzed one concentric circle around multiple Aβ plaques per section. The box plots demonstrated significant and differential changes in multiple proteins between 4 and 12 months that are related to the age-related deposition of amyloid and increased gliosis in response to pathology in the cortex (Figure 8A) and hippocampus (Figure 8B). More specifically, as expected, Aβ_42_ and APP levels are increased in 5XFAD mice compared to controls at 4 months, and these changes are further increased by 12 months in both the cortex (Figure 8A) and hippocampus (Figure 8B). Interestingly, within the cortex, there were few changes around the plaques between 5XFAD mice and controls with the exception of an increase in P2ry12 and a decrease in NeuN by 12 months in 5XFAD mice, likely due to previous observations of cell loss by 12 months in this model (Oakley et al., 2006; Jawhar et al., 2012; Eimer and Vassar, 2013; Figure 8A). Neuron loss has been qualitatively described in the subiculum by 9 months of age; in the neocortex, non-biased stereological estimates suggest that 5XFAD mice lose 25–40% of layer five pyramidal neurons between 9 and 12 months of age (Oakley et al., 2006; Jawhar et al., 2012; Eimer and Vassar, 2013). However, these mice do not develop a robust loss of neurons in the hippocampus (Oakley et al., 2006; Jawhar et al., 2012; Eimer and Vassar, 2013). However, by 12 months of age, the level of several protein markers associated with astrocytosis and microgliosis were elevated in the hippocampus of 5XFAD mice (Figure 8B). These data suggest that the cortex and hippocampus are differentially affected by amyloid deposition. These data are in line with our biochemical data and *in vivo* imaging data, as well as previously reported conventional histological staining (Oakley et al., 2006; Jawhar et al., 2012; Eimer and Vassar, 2013).

## Discussion

Therapeutic interventions are desperately needed for the treatment of AD, and the development of effective therapeutic strategies is limited by a lack of model organisms with disease characteristics sufficiently similar to that of human AD (Dodart et al., 2002; Duyckaerts et al., 2008; Citron, 2010; LaFerla and Green, 2012; Porquet et al., 2015; Jankowsky and Zheng, 2017). This likely contributes significantly to the challenges faced in translating animal-based findings to human therapies and, to date, no therapies have proven effective in clinical trials (Cavanaugh et al., 2014; Cummings et al., 2014, 2019; Jankowsky and Zheng, 2017). The MODEL-AD consortium aims to develop more translationally relevant AD mouse models in greater alignment with human AD and to enhance understanding of the phenotype of AD mouse models to facilitate enhanced translatability from mouse to human. Here, the IU/JAX/Pitt MODEL-AD Center conducted a deep phenotyping study of the commonly used 5XFAD mouse model, evaluating male and female mice aged 4, 6, and 12 months for overall frailty, cholesterol level, lipoprotein level, glucose metabolism, behavioral test response, sleep pattern, microbiome alteration, gene expression alteration, Aβ deposition, and proinflammatory marker expression.

We observed that 5XFAD mice exhibit age- and sex-based differences in total cholesterol, HDL, and LDL levels. Increasing evidence suggests that cholesterol levels may play an important role in AD development; specifically, low levels of HDL and elevated levels of LDL and total cholesterol are associated with increased risk for neurodegenerative diseases due to alterations in APP and Aβ accumulation, whereas high HDL levels offer protection (Kuo et al., 1998; Yaffe et al., 2002; Wolf et al., 2004; Eckert et al., 2010; Williamson and Sutherland, 2011; Gamba et al., 2012; Li et al., 2012; Posse de Chaves, 2012; Maulik et al., 2013). However, this association remains controversial as several studies have reported decreased risk for neurodegenerative disorders in the presence of elevated LDL (Reitz et al., 2004, 2008; Mielke et al., 2005), and another study indicated that cholesterol levels might be more important at presymptomatic stages than in late-stage disease (Pappolla et al., 2003). Indeed, to date, no benefits to patients with AD have been observed in any clinical trial involving cholesterol modulation with statins (Mejias-Trueba et al., 2018). The APOE ε4 allele is a major risk factor for LOAD development and functions as a regulator of cholesterol in the plasma and a cholesterol transporter in the brain (Saunders et al., 1993; de Chaves and Narayanaswami, 2008). There is a clear need for mouse models of AD in which cholesterol modulation in a setting such as that observed in human AD can be evaluated to further understanding of the true contribution of cholesterol levels to AD development and progression.

Furthermore, we evaluated sex differences in 5XFAD mice in comparison with WT controls at 4, 6, and 12 months of age. As cholesterol levels are known to differ based on sex in humans (Klingel et al., 2017), we compared male and female mice at all time points (Figures 1A–C) and noted a significant sex-based difference in total cholesterol in female mice in the 5XFAD and WT categories, demonstrating elevated levels in comparison with male 5XFAD and WT mice. Female 5XFAD and WT mice had higher LDL levels and lower HDL levels than males; however, again, no difference between 5XFAD and WT was observed, indicating that these alterations were not associated with the 5XFAD genotype. In males, LDL and HDL levels were significantly lower at 12 months of age than in 4- and 6-month-old mice (Figures 1B,C) and HDL levels decreased significantly between WT and 5XFAD mice at the 6- and 12-month time points, suggesting that at the later stage of disease, HDL decrease is associated with the 5XFAD genotype in male mice. Wirths et al. (2006) examined plasma cholesterol in APP/PS1 mice and found significant reductions in aged mice. These findings highlight potential challenges in evaluating cholesterol in the context of AD due to sex-based and age-based differences between mice that may be related to the 5XFAD genotype.

Increased frailty is a characteristic of AD in human patients (Song et al., 2011, 2014; Morley et al., 2013) and mouse models, and the level to which frailty is increased is an indicator of propensity to develop a more severe AD phenotype (Todorovic et al., 2020). MODEL-AD has validated the frailty measure used in the current studies, which is based on the presence and severity of a broad spectrum of characteristics associated with aging in mice, including changes in coat, body, and skin condition; ocular, nasal and oral complications; balance and gait impairments, and other age-related traits that are paralleled by the human aging process (Sukoff Rizzo et al., 2018). While the spectrum of aging changes tends to be subtle before 18–24 months of age in mice, there were notable early aging traits observed at 12 months of age in 5XFAD male and female mice that were not observed or expected in 12-month-old age-matched controls. This may be indicative of accumulating disease pathology in these mice. Consistent with observations in human AD (Theou et al., 2014), frailty scores were greater in female 5XFAD mice than in male mice at 6 and 12 months of age, indicating that the 5XFAD model recapitulates this sex-based divergence (Figure 2). Both male and female 5XFAD mice exhibited robust hyperactivity (Figure 2), consistent with previous characterizations of motor function in this model (Jawhar et al., 2012; O’Leary et al., 2018, 2020). As frailty and impairments in motor function are characteristics of human AD (Buchman and Bennett, 2011; Delrieu et al., 2016; Panza et al., 2018), these findings support the use of the 5XFAD model for investigating interventions directed toward improving physical condition and potentially impaired motor function in patients with AD; in particular, where motor function decline is observed in the preclinical stage of the disease (Buchman and Bennett, 2011).

However, despite previous reports that 5XFAD mice have cognitive deficits (Oakley et al., 2006; Jawhar et al., 2012), which may be specific to the particular cognitive assay being conducted, we did not see consistent cognitive impairment in 5XFAD mice on the C57BL/6J background at 6 or 12 months of age relative to age- and sex-matched controls in our assays. It is worth noting that our cognitive phenotyping was limited in scope given the recent recommendations by the NIA to prioritize more translational measures, which was the focus of our resources for this project. Given that 5XFAD mice are indeed hyperactive, we were also cognizant not to conduct cognitive assays such as water maze, novel object recognition, or fear conditioning that rely on motor activity and where hyperactivity is a major confound (Sukoff Rizzo et al., 2020; Vitek et al., 2020). Thus, we highly recommend that researchers interested in using 5XFAD mice as a model of robust plaque deposition also consider the confounds related to hyperactivity in any behavioral or cognitive assay chosen, especially for studies that will investigate the potential of novel compounds for preclinical efficacy. For a more translational biomarker of potential synaptic abnormalities that lead to cognitive deficits, we evaluated the EEG, which has also been used as a functional biomarker of mild cognitive impairment in patients with AD. Although an abnormal EEG was previously reported in 5XFAD mice on the hybrid background (Schneider et al., 2014; Siwek et al., 2015; Abe et al., 2020), we did not observe significant EEG abnormalities either as indicators of seizure activity, altered synaptic activity related to MCI, or sleep disturbance in this study. Therefore, we do not recommend the 5XFAD model for studies focused on sleep or seizure disturbances in AD or for translational studies evaluating the potential to improve cognitive deficits. This conclusion is supported by the lack of 18F-FDG findings in brain areas relevant for cognitive outcomes, despite alterations in glycolysis in brain regions associated with sensory integration and auditory function.

Furthermore, examination of the microbiome revealed that site-specific differences are the greatest source of microbiome variability. This could be due to multiple reasons, including housing conditions. Care should be taken when replicating studies between sites to account for site differences that may account for alterations in outcome measures.

Due to the known sex divergences in human AD and in the 5XFAD mouse model (Theou et al., 2014; Sohn et al., 2018; Arnold et al., 2020; O’Leary et al., 2020) and the critical need to consider the impact of sex differences in potential responses to treatments developed for AD (Nebel et al., 2018), we compared the brain transcriptome of male and female 5XFAD and WT mice, separating groups by genotype and sex with a principal component analysis to reveal significant sex- and genotype-disparities in gene expression (Figure 4). Previous work by Bouter et al. (2014) examined the transcriptome of two amyloid models (5XFAD and Tg4–42) to examine plaque-related and non-plaque related disease factors. Interestingly, Bouter et al. found a subset of neuroinflammation genes in 5XFAD female mice related to plaque deposition, similar to the present study, although the present study includes both males and females (Bouter et al., 2014). The group also identified DEGs that were identified in both amyloid models, suggesting disease pathways associated with behavioral deficits and neurodegeneration (Bouter et al., 2014). Future studies should include transcriptomics correlations with functional outcomes to identify subsets of genes associated with specific neurodegeneration processes. Other groups have looked at the transcriptome of the 5XFAD model and suggest that both frontal cortex and cerebellum in 5XFAD transgenic mice show specific pathological processes in the early pathophysiology of AD (Kim et al., 2012). In the present study and consistent with the increased prevalence of and risk for AD in human females versus males (Theou et al., 2014; Sohn et al., 2018; Arnold et al., 2020; O’Leary et al., 2020), genes with upregulated expression in 5XFAD versus WT mice were elevated in female 5XFAD mice to a greater extent than in male 5XFAD mice (Figure 4). Together, previous research and the current findings indicate that the 5XFAD mouse model is appropriate for studying sex differences in AD and for evaluating the influence of sex differences on the efficacy of potential AD interventions *in vivo*.

The AMP-AD consortium has developed a systems biology approach to characterizing changes in AD in which gene expression from AD and control brains were compared to build a profile of five Consensus Clusters of gene modules associated with AD (Logsdon et al., 2019). We compared gene expression changes in male and female 5XFAD versus WT mice at various ages with the AMP-AD Consensus Clusters and observed consistent gene expression changes across all five clusters in 5XFAD mice (Figure 4). Alterations were particularly pronounced in Consensus Cluster B, which consists of immune system pathways. As several studies have identified the immune system as a significant factor in AD development and progression (Gjoneska et al., 2015; Efthymiou and Goate, 2017), this pronounced alteration in immune system pathways in 5XFAD mice is consistent with the very early deposition of amyloid in this model and the subsequent response of the immune system due to the increased disease-related pathogenesis. Furthermore, in Consensus Cluster E, the majority of the changes observed were anti-correlated with human LOAD, suggesting that this model is not satisfactory for studying pathways associated with organelle biogenesis.

In addition to sex-based differences and gene expression changes, 5XFAD mice were also confirmed to express several features of AD pathology, demonstrating the model as useful for studies relying on the use of brain imaging and Aββ quantification strategies (Girard et al., 2013; Spencer et al., 2013; Macdonald et al., 2014; Tang et al., 2016; Kesler et al., 2018; Nie et al., 2019; Franke et al., 2020; Chang et al., 2021). Specifically, PET using [18F]-FDG and PET with [18F]-AV45 revealed significant uptake at all ages in both male and female mice (Figures 6, 7), demonstrating that [18F]-AV45 PET/MRI is feasible for quantifying Aβ in this model but should be confirmed with additional methods (Jankowsky and Zheng, 2017). Our [18F]-AV45 data are consistent with previous *in vivo* imaging studies in 5XFAD mice that looked at amyloid using a different tracer (18F-Florbetaben) and observed age-dependent increases in amyloid (Jankowsky and Zheng, 2017; Franke et al., 2020). This study also observed alterations in brain glucose metabolism and amyloid deposition prior to the onset of behavioral changes (Franke et al., 2020). Although previous studies detected cerebral hypometabolism (Franke et al., 2020), the methods for quantification, ROI and ages of animals were different in the present study. In addition, the animals in the present study did not undergo behavioral studies to monitor cognitive changes and PET imaging alterations. We utilized TissueVision’s imaging capabilities to map the deposition of amyloid throughout the brain over time. This is a powerful and useful tool for determining when to intervene in the treatment of amyloid deposition if using the 5XFAD model for preclinical studies. Future studies would benefit from correlating *in vivo* imaging studies with behavior and transcriptomics as a way to track the behavior, neurodegeneration and transcriptomic changes over time, similar to other work (Bouter et al., 2014; Franke et al., 2020). As further verification for the *in vivo* and *ex vivo* imaging studies, we confirmed that 5XFAD mice demonstrate significantly elevated soluble Aβ_40_ and Aβ_42_ at 4, 6, and 12 months of age in comparison with WT mice in the soluble fraction of the hemibrain and the plasma (Supplementary Figure 2), recapitulating findings in human AD and indicating that 5XFAD mice are appropriate for studies focused on Aβ_40_ and Aβ_42_ accumulation.

Neuroinflammatory changes in the brain by gene expression matched inflammatory changes in the plasma and brain (Supplementary Figure 2). Importantly, 5XFAD mice develop an age-dependent increase in amyloidosis, neuron cell loss, and neuroinflammation, as evidenced by NanoString protein analysis, which is consistent with human AD and has been previously reported in this model (Oakley et al., 2006; Jawhar et al., 2012; Eimer and Vassar, 2013; Figure 8).

In summary, we conducted a deep phenotyping study comparing 5XFAD to WT mice that revealed that 5XFAD mice recapitulate human AD in the context of (1) frailty and abnormal motor function, (2) sex-based alterations in condition and gene expression, (3) immune system involvement in disease development and (4) amyloidosis. This rigorous pipeline enables researchers conducting preclinical studies to make decisions on the types of studies the 5XFAD mouse model is appropriate for as well as which treatments to test in the 5XFAD model based on a drug’s mechanism of action and the model’s disease trajectory. Therefore, we recommend the use of 5XFAD mice for AD studies focused on sex-based differences, immune regulation, and Aβ deposition but not for translational studies evaluating the potential of therapeutics to treat cognitive impairments. Further, the pipeline described herein and applied to the comprehensive phenotyping of the 5XFAD mice will be used for future studies to comprehensively characterize new models of LOAD being generated as part of our MODEL-AD efforts.

## Data Availability Statement

The original contributions presented in the study are publicly available. The molecular, bioinformatics and imaging data are available via the AD Knowledge Portal: https://adknowledgeportal.org (permission was obtained for this material through a Creative Commons CC-BY license). The data, analyses, and tools are shared early in the research cycle without a publication embargo on secondary use. Data are available for general research use according to the following requirements for data access and data attribution (https://adknowledgeportal.org/DataAccess/Instructions). To access the content described in this article, see: https://doi.org/10.7303/syn23638028.

## Ethics Statement

The animal study was reviewed and approved by Indiana University School of Medicine IACUC, The Jackson Laboratory Animal Use Committee, and University of Pittsburgh IACUC.

## Author Contributions

AO, KK, ZC, AB, BAL, SS, PT, GC, GH, MS, and BTL contributed to conception and design of the study. AO organized the data and wrote the first draft of the manuscript. AO, KK, ZC, AB, BAL, SS, PT, GC, and RP performed the statistical analysis. RP, ZC, AB, SS, PT, and MS wrote sections of the manuscript. All authors contributed to manuscript revision, read, and approved the submitted version.

## Conflict of Interest

TR and AB are employees of TissueVision, Inc. TR is a shareholder of TissueVision, Inc. The remaining authors declare that the research was conducted in the absence of any commercial or financial relationships that could be construed as a potential conflict of interest.

## Publisher’s Note

All claims expressed in this article are solely those of the authors and do not necessarily represent those of their affiliated organizations, or those of the publisher, the editors and the reviewers. Any product that may be evaluated in this article, or claim that may be made by its manufacturer, is not guaranteed or endorsed by the publisher.

## Footnotes

1
https://adknowledgeportal.synapse.org/

## Supplementary Material

Supplementary Figure 1 The largest diversity of microbiome is site. Mice from Indiana University, The Jackson Laboratory, and the University of Pittsburgh were assessed between 2 and 21 months of age.

Supplementary Figure 2 Aβ and proinflammatory cytokines are upregulated in 5XFAD brain and plasma. Significant increases in both soluble Aβ_40_ and Aβ_42_ were observed at all time points in both hemibrain **(A)** and plasma **(B)**. In addition, significantly increased proinflammatory markers were found in hemibrain (IL-β, TNF-α) **(C)**.

Supplementary Table 1 Table of methoxy-X04 labeled plaque densities across 839 total brain regions in the Allen Brain Reference Atlas for all animals (2 months – eight females, eight males; 3 months – seven females, eight males; 4 months – eight females, six males; 6 months – eight females, five males). Table columns denote sample (sample id), brain region (structure_name), total voxels in structure (structure_voxels), total masked plaque voxels (plaque_voxels), plaque density (plaque_density), sample age in months (age_months), genotype (genotype), and sex (sex).
