# Supplementary figures and images for: Comprehensive Evaluation of the 5XFAD Mouse Model for Preclinical Testing Applications: A MODEL-AD Study
Source: Front Aging Neurosci. 2021 Jul 23;13:713726. doi: 10.3389/fnagi.2021.713726 (PMC8346252; doi:10.3389/fnagi.2021.713726)

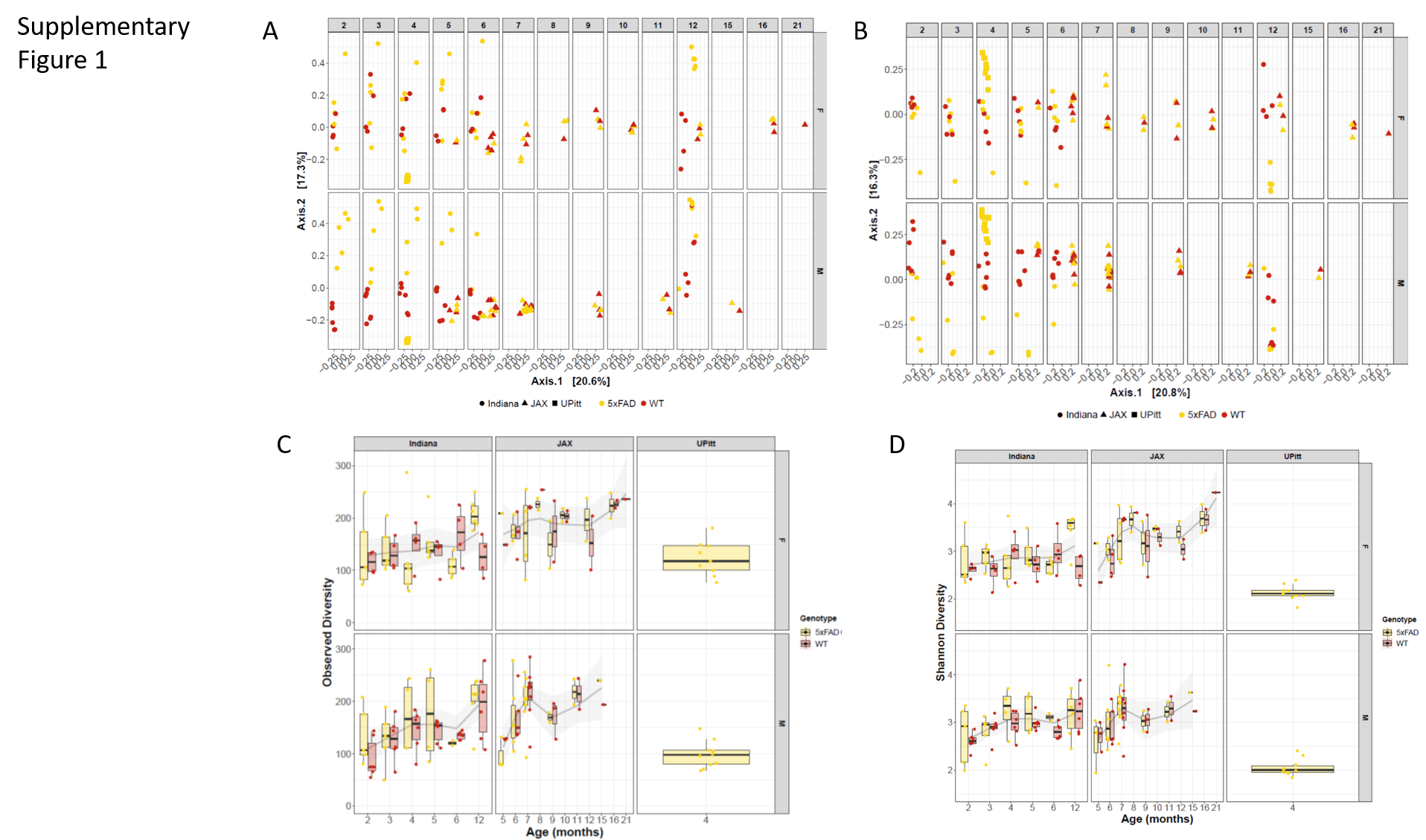

Supplement: Supplementary Figure 1 — The largest diversity of microbiome is site. Mice from Indiana University, The Jackson Laboratory, and the University of Pittsburgh were assessed between 2 and 21 months of age. [file Image_1.TIF]

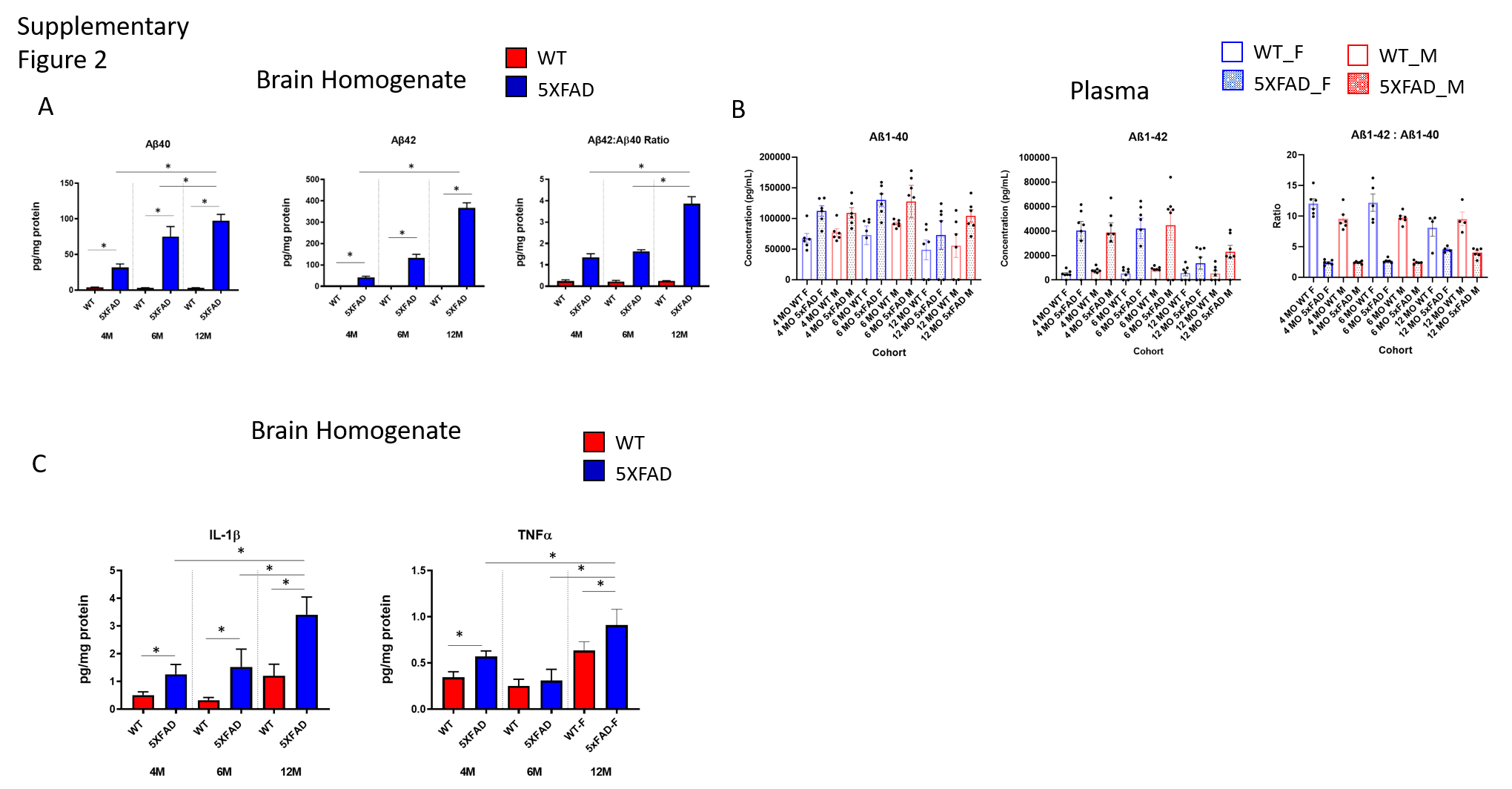

Supplement: Supplementary Figure 2 — Aβ and proinflammatory cytokines are upregulated in 5XFAD brain and plasma. Significant increases in both soluble Aβ40 and Aβ42 were observed at all time points in both hemibrain (A) and plasma (B). In addition, significantly increased proinflammatory markers were found in hemibrain (IL-β, TNF-α) (C). [file Image_2.TIF]
